# Pylephlebitis and Right-Sided Endocarditis: A Rare Complication of an Intra-abdominal Infection

**DOI:** 10.7759/cureus.59372

**Published:** 2024-04-30

**Authors:** Lyndon Sprenghers, Lode Van Overbeke, Christophe Libeer

**Affiliations:** 1 Internal Medicine, KU Leuven, Leuven, BEL; 2 Gastroenterology, AZ Sint-Maarten, Mechelen, BEL; 3 Pulmonology, AZ Sint-Maarten, Mechelen, BEL

**Keywords:** streptococcus bovis, streptococcus gallolyticus, tricuspid valve endocarditis, right-sided endocarditis, pylephlebitis

## Abstract

Right-sided infective endocarditis is less common than left-sided endocarditis and can be a difficult clinical diagnosis. The presence of intracardiac devices is a major risk factor. The presentation is less clear than left-sided forms because of the presence of respiratory symptoms and the absence of systemic embolization. Pylephlebitis, or septic thrombosis of the portal vein, is a serious infectious condition that often delays diagnosis. It is a complication of intraabdominal or pelvic infections. Streptococcus gallolyticus (S. gallolyticus) can cause infective endocarditis and is associated with colon neoplasia and hepatobiliary disease.

In this case report, we describe the case of a 76-year-old male with a history of rectal adenocarcinoma who presented with different episodes of fever of unknown origin (FUO), one of which occurred after pacemaker implantation. Ultimately, he was diagnosed with S. gallolyticus-mediated tricuspid valve endocarditis with underlying pylephlebitis. Investigations did not show evidence of pacemaker lead endocarditis.

## Introduction

Right-sided infective endocarditis is less common than left-sided endocarditis and accounts for 5-10% of all cases. Known risk factors are IV drug use, right-sided cardiac anomalies, and the presence of intravascular or cardiac devices [1]. The presentation is less clear than left-sided forms because of the presence of respiratory symptoms and the absence of systemic embolization. The most frequent isolated pathogen is Staphylococcus aureus. Most cases resolve completely with appropriate antibiotic therapy and have a favorable prognosis [2].

Following Staphylococcus aureus (S. aureus), streptococci and enterococci are the second and third most common pathogens. Streptococci account for 5-30% of the cases. Streptococcus gallolyticus (S. bovis biotype I) (S. gallolyticus) is one of these pathogens [3]. S. gallolyticus can cause infective endocarditis and is associated with colon neoplasia and hepatobiliary disease [4]. The mechanism of this association with colon neoplasia is not completely understood, but the association only exists for S. bovis biotype I [5]. Because of this association, the American Heart Association and Infectious Diseases Society of America guidelines recommend performing a colonoscopy in patients with S. gallolyticus bacteremia.

Pylephlebitis, or septic thrombosis of the portal vein, is a serious infectious condition that often delays diagnosis. It has an incidence of 0.37-2.7 cases per 100,000 person-years [6].

It is a complication of intra-abdominal or pelvic infections and develops because the region of infection is drained by the portal venous system [7]. Bacteraemia associated with this condition is often polymicrobial. The disease usually presents with an acute onset of fever and abdominal pain in the right upper quadrant. Diagnosis is made with blood cultures and radiography (most frequently abdominal CT scan) [8]. We present the case of a S. gallolyticus-mediated tricuspid valve endocarditis with underlying pylephlebitis.

## Case presentation

Patient description

**Figure 1:**
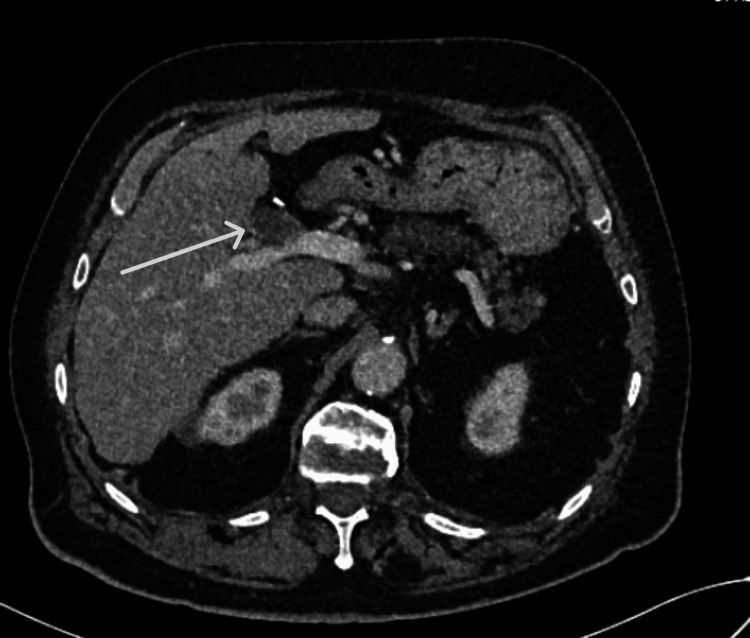
CT scan of the abdomen showing the initial left portal vein thrombosis

We present the case of a 76-year-old caucasian male. In 2012, he was diagnosed with rectal adenocarcinoma, for which he was treated with neoadjuvant chemoradiotherapy and, ultimately, an ultra-low total mesorectal excision (TME). Surgery was complicated with perforation of the colon with secondary fecal peritonitis, intestinal ischemia, and septic shock with multiple organ failure. Because of this complicated course, the patient received a permanent colostomy, and neoadjuvant therapy was started. In 2021, our patient had cholangitis based on choledocholithiasis. After treatment of the cholangitis, a cholecystectomy was performed. The man developed a partial portal vein thrombosis of the left portal vein branch secondary to the cholangitis, and there were no signs of secondary portal hypertension. Apixaban was started. Janus kinase 2 (JAK2) screening for possible essential thrombocytosis was negative. To our knowledge, other screenings for thrombophilia have not been performed. After six months of anticoagulation, a follow-up abdomen CT was performed; this showed no changes in the partial portal vein thrombosis (Figure 1). Apixaban was continued for another six months. After twelve months of anticoagulation, an abdomen CT showed no recanalization of the left portal vein branch. Apixaban was stopped because there was a clear provoking factor of the thrombosis (secondary to an episode of cholangitis), and recanalization was no longer expected after twelve months of anticoagulation.

Besides the oncological history and the cholangitis with partial portal vein thrombosis, the man was also known to have chronic kidney disease with a baseline creatinine clearance of 35ml/min.

More recently, in October and November 2022, the man was hospitalized twice in our ward. In October, he presented with interscapular pain and dyspnoea for one day. The patient denied any fever, but at the emergency department, he was subfebrile. Inflammatory markers were elevated. CT of the chest and abdomen with IV contrast showed bilateral ground glass opacities without other abnormalities. The diagnosis of beginning pneumonia was made, and the patient received seven days of amoxicillin-clavulanic acid with good effect clinically as well as biochemically. During this first admission, no blood cultures were taken at the emergency department; blood cultures taken after the start of antibiotic therapy remained sterile.

In November, he presented again at the emergency department because of palpitations, light-headedness, and malaise for three days. CRP was elevated again, and clinically, no focus was found. Because of the recent hospitalization, piperacillin-tazobactam was started after blood cultures were taken. During this admission, he had no fever, and cultures taken after the start of antibiotics remained sterile. He received antibiotics for five days. During this hospitalization, a transoesophageal echocardiogram (TEE) was negative, and no other imaging was performed because of the absence of a clinical focus and a favorable clinical and biochemical evolution. There was no clear working diagnosis for this episode of inflammation. The man developed tachy-brady syndrome during the hospitalization. He received a pacemaker after antibiotic treatment (five days of piperacillin-tazobactam). In both episodes, full recovery was seen clinically with a favorable biochemical evolution. The evolution of CRP with the dates of the different admissions (Table 1). In December 2022, the man presented a fever for a third time to the emergency department. In the next sections, we will discuss the course and findings of this case.

**Table 1:** Evolution of CRP during the three admissions with medical information about cultures and antibiotics CRP: C-reactive protein

Date (DD/MM/YYYY)	CRP in mg/L	Medical information
23/10/2022	51	First admission (23/10/2022 – 26/10/2022). No hemocultures taken on admission, hemocultures after start of antibiotics remained negative. 7 days of amoxicillin-clavulanic acid
24/10/2022	181.4	
26/10/2022	138	
28/11/2022	104.1	Second admission (28/11/2022 – 05/12/2022). Hemocultures taken before the start of antibiotics, all cultures remained negative. 5 days of piperacillin-tazobactam. 02/12/2022 pacemaker implantation (day 5 of antibiotics)
01/12/2022	37.1	
03/12/2022	21.4	
20/12/2022	104.9	Third admission (20/12/2022 – 03/01/2023). Hemocultures positive for S. gallolyticus. Subject of the case report.
21/12/2022	148.8	
23/12/2022	62.4	
26/12/2022	20.3	
28/12/2022	9.7	
02/01/2023	5.2	
12/01/2023	8.2	Follow-up cardiology. Full recovery clinically as well as biochemically. Echocardiography was normal.
20/01/2023	9.0	
07/02/2023	5.8	
11/05/2023	8.9	

Case history

The patient had been discharged on December 5th from the second admission; on December 20th, he presented again at the emergency department.

He suffered from fever (up to 38.8°C) and chills for three days. He had been fatigued and was feeling ill. For the last few days, there was anorexia without any other gastrointestinal complaints. The patient had been completely free of symptoms between the different hospitalizations. The remaining history was completely negative.

Physical examination results

We reported a high-grade fever of 40.3°C with chills at the emergency department. The other vital signs were within normal limits. The man did not look acutely ill, and he was normally oriented. Cardiac and pulmonary examinations were normal, and there were no heart murmurs. Abdominally, we withheld pain on palpation of the right upper quadrant with a positive Murphy's sign. The remainder of the physical examination was normal.

Results of pathological tests and other investigations

**Figure 2:**
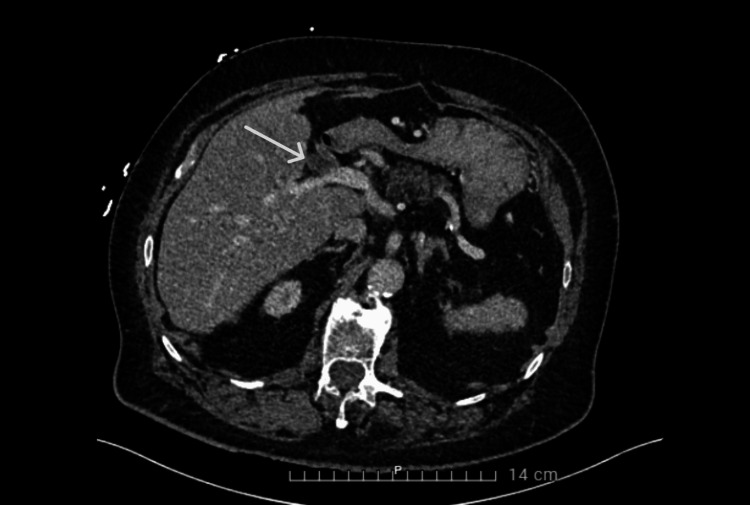
CT scan of the abdomen showing the known left portal vein thrombosis

Laboratory results reported a CRP of 104.9 mg/L (reference <5mg/L), leucocytosis with left shift (14.03x10³/µL, reference 3.64-8.46x10³/µL), and a mild elevation of gamma-glutamyl transferase (146 U/L, reference ≤ 55 U/L) and alkaline phosphatase (167 U/L, reference 50-116 U/L) which was already known for a longer time in the context of liver steatosis. Chest X-ray was normal. An abdominal CT scan with IV contrast showed the known portal vein thrombosis without other abnormalities (Figure 2).

**Figure 3:**
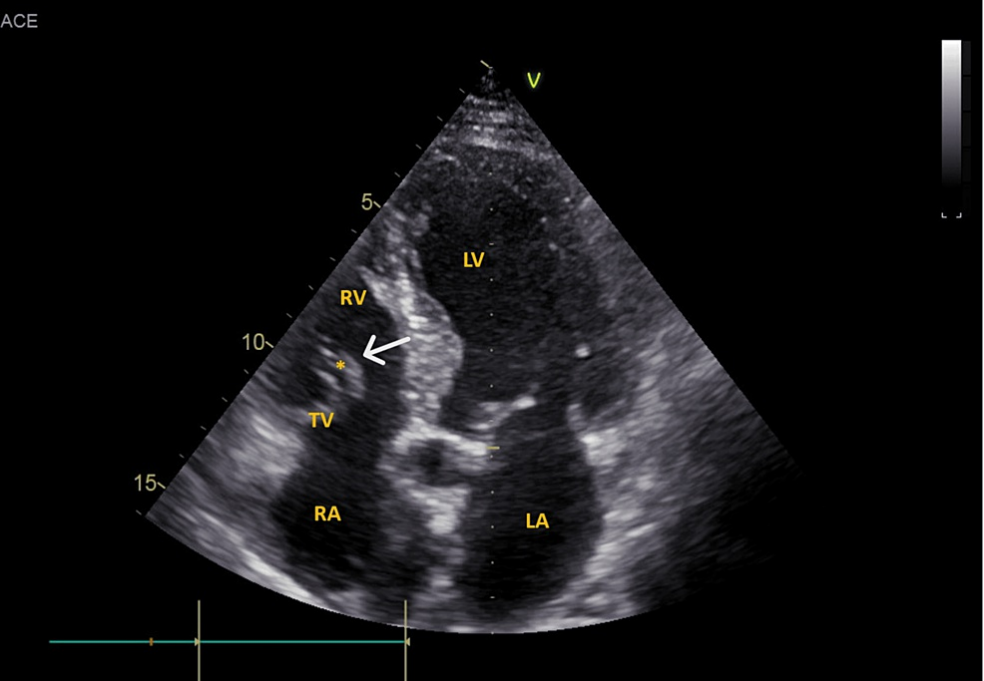
TTE showing a mobile mass on the tricuspid valve RV: right ventricle; LV: left ventricle; LA: left atrium; RA: right atrium; TV: tricuspid valve; *: added structure/vegetation; TTE: transthoracic echocardiogram

To minimize potential nephrotoxicity, intravenous saline prehydration was administered (the patient was known to have chronic kidney disease). Cultures were taken, and blood cultures were positive for Streptococcus gallolyticus (S. bovis biotype I); other cultures remained negative. A transthoracic echocardiogram (TTE) showed a mobile mass on the tricuspid valve, suggesting vegetation (Figure 3).

**Figure 4:**
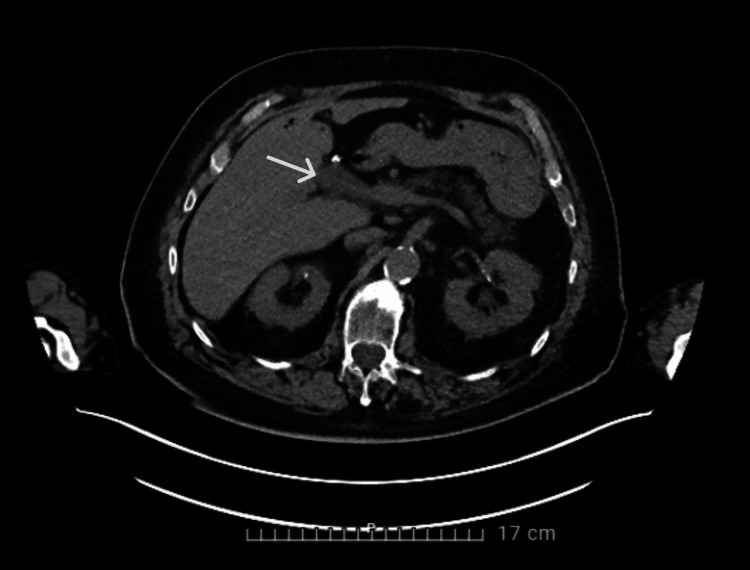
CT of the abdomen showing distension of the portal vein with surrounding soft tissue cuff

**Figure 5:**
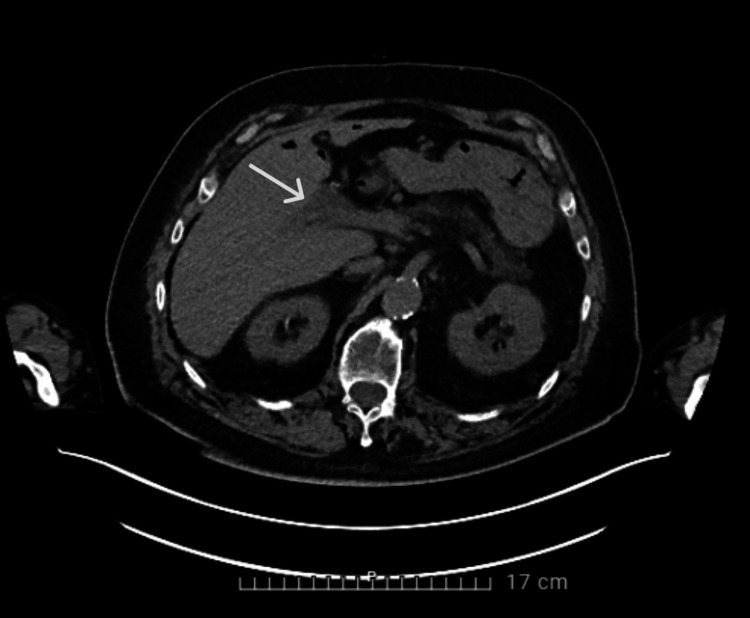
CT scan of the abdomen showing thrombophlebitis of the portal vein

TEE was performed for better visualization; this showed no vegetation on the pacemaker leads. A possible vegetation was visualized on the tricuspid valve; however, it was less clearly visible than on TTE. A positron emission tomography-computed tomography (PET-CT) was performed because there was no clear focus for the bacteremia and endocarditis; an abdominal focus was suspected because of the S. gallolyticus. The CT showed mild distension of the portal vein with limited surrounding soft tissue cuff, compatible with thrombophlebitis of the portal vein (Figure 4-5).

**Figure 6:**
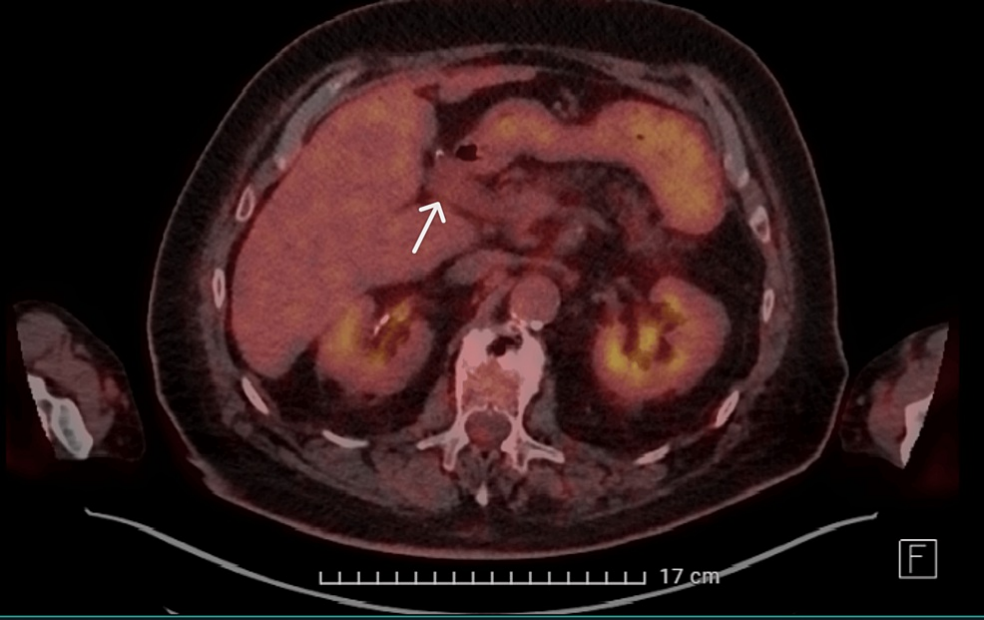
PET scan showing slightly increased metabolic activity at the portal vein and the surrounding soft tissue cuff PET: positron emission tomography

**Figure 7:**
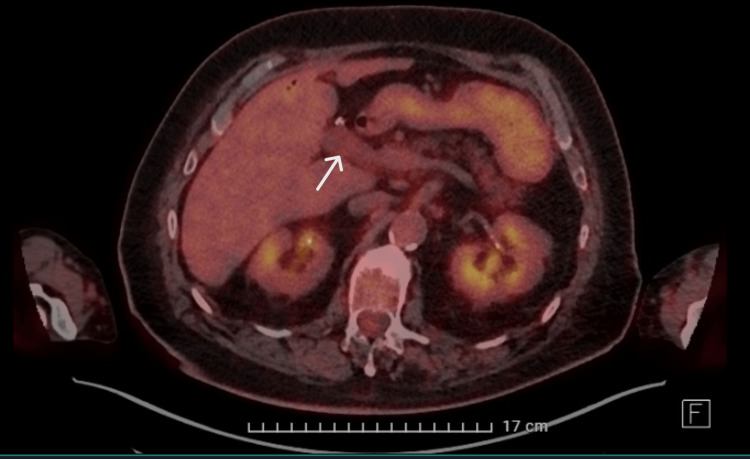
PET scan showing only limited increased metabolic activity at the portal vein PET: positron emission tomography

PET showed only slightly increased metabolic activity at the portal vein; there was no other clear focus for the bacteremia (Figure 6, 7). PET showed no abnormalities consistent with endocarditis.

Based on these investigations, the diagnosis of tricuspid valve endocarditis originating from portal vein thrombophlebitis (pylephlebitis) was made.

Treatment plan

Because of the high fever and the recent hospitalizations, the patient was started on piperacillin-tazobactam; this antibiotic was previously used with good results. Based on the antibiogram, the antibiotic therapy was then switched to ceftriaxone. Blood cultures were repeatedly taken but remained sterile after the start of antibiotic therapy. After a multidisciplinary discussion with cardiology, gastroenterology, and microbiology, we decided to continue ceftriaxone for four weeks. After thirteen days of intravenous therapy, antibiotic therapy was continued via outpatient parenteral antimicrobial therapy (OPAT) because of the good general condition of the man.

Expected outcome of the treatment plan

We expected the patient to be fever-free after starting the right antibiotics. We expected the control blood cultures to remain sterile and the inflammation to disappear.

Actual outcome

The patient was indeed fever-free after the start of the antibiotic treatment. Subsequent blood cultures were sterile, and we saw a favorable evolution of the inflammation. Our patient was discharged with OPAT and was closely followed by the cardiology department. Follow-up TTE could not visualize any vegetation. After four weeks, ceftriaxone was stopped. After stopping antibiotic therapy, the patient remained fever-free, and blood work remained normal. Even after several months, the inflammatory markers remained low. Table 1 shows the evolution of CRP. A colonoscopy was performed after the initial treatment of the infection, and it was completely normal.

## Discussion

In this case, the diagnosis of infective endocarditis was not hard to make; blood cultures were positive for S. gallolyticus, and the first echocardiography already showed vegetation at the tricuspid valve. The hospitalization had been preceded by two more hospitalizations with inflammation of unknown origin. The first time, a diagnosis of beginning pneumonia was made; the second time, no focus was found. The question arises whether these two episodes were already part of the S. gallolyticus bacteremia. Blood cultures at the time were negative. However, in the first hospitalization, antibiotics were started before the blood cultures were collected. We know there was no infective endocarditis at that time because a TEE was performed and did not show any vegetation.

During the second hospitalization, we considered the use of a PET scan because it was already the second episode of inflammation without a clear focus; we did not perform it because of the quick recovery of the patient on antibiotics. Retrospectively, it is hard to say whether fluorodeoxyglucose-positron emission tomography (FDG-PET) would have been useful in this case because of the negative blood cultures and the subtle FDG uptake we saw on PET during the third hospitalization. Studies on the role of FDG-PET in identifying causes of fever of unknown origin (FUO) and inflammation of unknown origin (IUO) show a more than 50% diagnostic yield. The clinical and biochemical factors influencing the likelihood of a diagnostic FDG-PET/CT are unclear. Some studies show that CRP and leukocytes do not predict a diagnostic PET/CT outcome [9]. In other studies, the likelihood of a diagnostic FDG-PET/CT was increased in higher age (>50 years) and elevated CRP [10]. Although it is already common practice, more studies on the role of FDG-PET in cases of FUO/IUO are needed.

As discussed in the introduction, the presence of intracardiac devices is a risk factor for right-sided endocarditis. During the second episode of inflammation, our patient developed a tachy-brady syndrome with significant sinus pauses. After 5 days of piperacillin-tazobactam, a pacemaker was implanted. Although the pacemaker was inserted shortly after the inflammatory episode, we do not have any indications that it was implanted during an episode of bacteremia, given the negative blood cultures and the decline in CRP levels during antibiotic therapy (Table 1). The latter does, however, suggest the inflammation was part of an infectious condition rather than a non-infectious condition.

Right-sided infective endocarditis is associated with intracardiac and intravenous devices. In this case, no vegetation was seen on the pacemaker leads, but the presence of an intracardiac device can facilitate the development of vegetation on the right side of the heart. The first 6 to 12 months after pacemaker implantation has a 0,5-1% risk of infective endocarditis [11].

Given the important association between S. gallolyticus and colon neoplasia, guidelines do recommend performing a colonoscopy. Our patient was already diagnosed and treated for rectum carcinoma and was in follow-up with a gastroenterologist; the last colonoscopy was performed in 2020 and was normal. A new colonoscopy was planned in May 2023 and was also completely normal [12]. The guideline does not specify when to perform the colonoscopy, so we decided to first treat the infective endocarditis.

In this case, the presence of pylephlebitis was discovered on PET-CT. Pathophysiologically speaking, it is not really a pylephlebitis. A pylephlebitis develops, as discussed when an intra-abdominal or pelvic infection is drained by the portal venous system. Our patient already had a partial portal vein thrombosis, which occurred during an episode of cholangitis. At the time of presentation, he no longer received anticoagulants, Apixaban was stopped after 12 months, as discussed earlier. After implantation of the pacemaker, Apixaban was started again because of the atrial fibrillation (tachy-brady syndrome). So, most likely, the portal vein thrombosis was infected secondary to the bacteremia caused by bacterial translocation from the gastrointestinal tract. CT scan showed an image compatible with thrombophlebitis of the portal vein, and PET showed only moderate metabolic activity. The diagnosis of pylephlebitis is made with blood cultures and mostly CT scans. In addition, our patient also suffered pain in the upper right quadrant. The role of PET in diagnosing pylephlebitis has not yet been studied. We found one case report in which PET aided in making the diagnosis [13].

## Conclusions

We presented the case of a 76-year-old male who developed S. gallolyticus bacteremia with secondary pylephlebitis and tricuspid valve endocarditis. We think it is important to repeat diagnostic studies when something changes in the presentation (e.g., during the second inflammatory episode, TEE was negative; it was repeated because of new positive blood cultures and eventually showed tricuspid valve endocarditis). PET-CT is increasingly important in fever/inflammation of unknown origin and plays an important role in the diagnostics of this case. Lastly, it is important to rule out colon neoplasia when confronted with S. gallolyticus. A colonoscopy should be performed.
